# THE UNIQUE CONNECTION BETWEEN PERSONALITY TRAITS AND LONELINESS AMONG CENTENARIANS

**DOI:** 10.1093/geroni/igz038.150

**Published:** 2019-11-08

**Authors:** Jinmyoung Cho, Alex J Bishop

**Affiliations:** 1 Baylor Scott & White Health, Temple, Texas, United States; 2 Oklahoma State University, Stillwater, Oklahoma, United States

## Abstract

Evidence suggests that personality traits contribute to risk and resilience in long-term survivorship. However, research examining the link between personality and loneliness among persons living 100 and more years has remained limited. The purpose of this study was to examine the association between Big 5 personality traits and loneliness among 154 centenarians residing in Oklahoma. Basic descriptive and multivariate regression analyses were conducted. Analyses of covariance (ANCOVA) indicated that mean level of loneliness was higher among centenarians possessing higher levels of extraversion, agreeableness, conscientiousness, and lower level of neuroticism compared to their counterparts. After controlling for demographic characteristics, physical health and cognitive functioning, neuroticism (β=-.22, p<.05) and agreeableness (β=.40, p<.001) were significantly associated with loneliness. It appears that experiencing emotional instability and being agreeable contributes to greater feelings of loneliness among centenarians. This has implications relative to further investigating how personality may uniquely contribute to loneliness after age 100.

